# Hypoxia mimetic activity of VCE-004.8, a cannabidiol quinone derivative: implications for multiple sclerosis therapy

**DOI:** 10.1186/s12974-018-1103-y

**Published:** 2018-03-01

**Authors:** Carmen Navarrete, Francisco Carrillo-Salinas, Belén Palomares, Miriam Mecha, Carla Jiménez-Jiménez, Leyre Mestre, Ana Feliú, Maria L. Bellido, Bernd L. Fiebich, Giovanni Appendino, Marco A. Calzado, Carmen Guaza, Eduardo Muñoz

**Affiliations:** 1 Vivacell Biotechnology SL, Córdoba, Spain; 2 Departamento de Neurobiología Funcional y de Sistemas, Instituto Cajal-CSIC, Madrid, Spain; 3 Instituto Maimónides de Investigación Biomédica de Córdoba (IMIBIC), University of Córdoba, Avda Menéndez Pidal s/n, 14004 Córdoba, Spain; 4 Departamento de Biología Celular, Fisiología e Inmunología, Universidad de Córdoba, Córdoba, Spain; 5 Hospital Universitario Reina Sofía, Córdoba, Spain; 6 Emerald Health Pharmaceuticals, San Diego, CA USA; 7 Vivacell Biotechnology GmbH, Denzlingen, Germany; 8 Dipartimento di Scienze del Farmaco, Università del Piemonte Orientale, Novara, Italy

**Keywords:** Multiple sclerosis, Cannabinoids, PHDs, HIF-1α, VEGF, EPO, Arginase 1

## Abstract

**Background:**

Multiple sclerosis (MS) is characterized by a combination of inflammatory and neurodegenerative processes variously dominant in different stages of the disease. Thus, immunosuppression is the goal standard for the inflammatory stage, and novel remyelination therapies are pursued to restore lost function. Cannabinoids such as ^9^Δ-THC and CBD are multi-target compounds already introduced in the clinical practice for multiple sclerosis (MS). Semisynthetic cannabinoids are designed to improve bioactivities and druggability of their natural precursors. VCE-004.8, an aminoquinone derivative of cannabidiol (CBD), is a dual PPARγ and CB_2_ agonist with potent anti-inflammatory activity. Activation of the hypoxia-inducible factor (HIF) can have a beneficial role in MS by modulating the immune response and favoring neuroprotection and axonal regeneration.

**Methods:**

We investigated the effects of VCE-004.8 on the HIF pathway in different cell types. The effect of VCE-004.8 on macrophage polarization and arginase 1 expression was analyzed in RAW264.7 and BV2 cells. COX-2 expression and PGE_2_ synthesis induced by lipopolysaccharide (LPS) was studied in primary microglia cultures. The efficacy of VCE-004.8 in vivo was evaluated in two murine models of MS such as experimental autoimmune encephalomyelitis (EAE) and Theiler’s virus-induced encephalopathy (TMEV).

**Results:**

Herein, we provide evidence that VCE-004.8 stabilizes HIF-1α and HIF-2α and activates the HIF pathway in human microvascular endothelial cells, oligodendrocytes, and microglia cells. The stabilization of HIF-1α is produced by the inhibition of the prolyl-4-hydrolase activity of PHD1 and PDH2. VCE-004.8 upregulates the expression of HIF-dependent genes such as erythropoietin and VEGFA, induces angiogenesis, and enhances migration of oligodendrocytes. Moreover, VCE-004.8 blunts IL-17-induced M1 polarization, inhibits LPS-induced COX-2 expression and PGE_2_ synthesis, and induces expression of arginase 1 in macrophages and microglia. In vivo experiments showed efficacy of VCE-004.8 in EAE and TMEV. Histopathological analysis revealed that VCE-004.8 treatments prevented demyelination, axonal damage, and immune cells infiltration. In addition, VCE-004.8 downregulated the expression of several genes closely associated with MS physiopathology, including those underlying the production of chemokines, cytokines, and adhesion molecules.

**Conclusions:**

This study provides new significant insights about the potential role of VCE-004.8 for MS treatment by ameliorating neuroinflammation and demyelination.

**Electronic supplementary material:**

The online version of this article (10.1186/s12974-018-1103-y) contains supplementary material, which is available to authorized users.

## Background

Multiple sclerosis (MS), a chronic autoimmune demyelinating disease of the central nervous system (CNS), is one of the most common acquired neurological diseases in young adults. The hallmarks of MS include neuroinflammation, caused by the migration of leukocyte infiltrates into the CNS, and loss of myelin and axonal damage [1]. Disease progression is considered the result of two related processes, namely myelin destruction (demyelination) with failure to remyelinate [2] and progressive axonal damage with little capacity for recovery [3]. Remyelination is an endogenously regulated process orchestrated by the generation of new mature oligodendrocytes. These cells provide new myelin sheathes to demyelinated axons, promoting the recovery of axonal integrity and functional deficits [4, 5]. Exacerbated innate and adaptative immune responses contribute to the physiopathology of the disease, and the majority of current therapies for MS are directed towards modulation of the immune response [6]. However, novel therapies aimed to axonal remyelination are urgently needed.

Hypoxia preconditioning induced by mild oxygen depletion is beneficial in a wide number of neurological disorders including MS [7, 8]. The cellular adaptation to severe or mild hypoxia is very fast and involves the activation of the hypoxia-inducible factor (HIF), an ubiquitous transcription factor that accumulates in response to hypoxia and regulates a plethora of genes involved in many biological processes including erythropoiesis, angiogenesis, vascular tone, and immunity [9, 10].

HIF-1α activation may play a role in the inflammatory and the remitting phases of MS (reviewed by [11]). For instance, HIF-1α may exert anti-inflammatory activity by inducing the release of TGFβ [12, 13], a potent anti-inflammatory cytokine, and by upregulating the FoxP3 gene implicated in the differentiation of Tregs [14]. In addition, there is evidence suggesting that activation of the HIF pathway may be also linked to neuroprotection and perhaps remyelination [15]. Thus, the erythropoietin (EPO) gene is HIF-dependent, and EPO is neuroprotective in different animal models of MS [16, 17]. In addition, methylprednisolone, a widely used glucocorticoid for treating MS, protects oligodendrocytes from excitotoxicity through a HIF-1α-dependent pathway [18]. Indeed, HIF-1α stabilization protects oligodendrocytes against TNF-α-mediated cell death [15].

On the other hand, HIF-1α activates several proangiogenic genes including vascular endothelial growth factor (VEGF-A) and fibroblast growth factor-2, which are mainly produced by vascular endothelial cells. The vascular endothelial cells produce trophic factors to maintain brain homeostasis within the context of the neurovascular unit [19, 20]. Accordingly, signaling network acting between cerebral endothelium and neuronal precursor cells is responsible for sustaining neurogenesis and angiogenesis even in the adult brain [19–24]. In the case of white matter, it has been proposed that a corresponding “oligovascular niche” may also exist, wherein cerebral endothelial cells promote the proliferation and migration of oligodendrocyte precursor cells (OPCs) [25, 26]. OPC might recognize their path to migrate along the vasculature, and it has been shown recently that OPC-endothelial interaction occurs by Wnt-Cxcr4 signaling, at least in the developing CNS and in postnatal spinal cord [27].

VCE-004.8 is an aminoquinone derivative of cannabidiol endowed with dual PPARγ and CB_2_ activity [28]. Both endpoints are druggable targets for MS, and we show here that VCE-004.8 also targets the HIF pathway, expanding the rationale for its development as a novel MS drug.

## Methods

### Cell lines and reagents

Murine RAW264.7 (ATCC^®^ TIB-71^™^) and BV-2 cell lines were maintained at 37 °C in a humidified atmosphere containing 5% CO_2_ in DMEM supplemented with 10% fetal calf serum (FCS), 2 mM l-glutamine, and 1% (*v*/*v*) penicillin/streptomycin. Human brain microvascular endothelial cells (HBMEC) were maintained in endothelial cell medium (ScienCell, San Diego, CA, USA) supplemented with 5% FBS, 1% ECGS, and 1% penicillin/streptomycin. Human dermal microvascular endothelial cells (HMEC-1) (ATCC^®^ CRL-3243^™^) were maintained in MCDB131 medium (Life Technologies, Carlsbad, CA, USA) supplemented with 10 ng/ml epidermal growth factor (EGF), 1 μg/ml hydrocortisone, 10 mM glutamine, and 10% FBS. The transformed human microglial cells (HMC3) (ATCC^®^ CRL-3304^™^) were maintained in Eagle’s minimum essential medium supplemented with 10% FBS and 1% penicillin/streptomycin. Human oligodendrocyte cell line MO3.13 was obtained from Tebu-Bio (Barcelona, Spain) and was cultured in complete medium containing DMEM supplemented with 10% fetal bovine serum (FBS) and 1% penicillin/streptomycin (P/S) at 37 °C, 5% CO_2_. Cells were allowed to differentiate for 3 days further by replacing the complete medium with serum-free DMEM supplemented with 100 nM PMA and 1% P/S. Differentiated cells are positive for markers such as myelin basic protein (MBP) and myelin oligodendrocyte glycoprotein (MOG), which are phenotypic markers of mature, myelinating oligodendrocytes. The mouse NIH3T3-EPO-luc cells have been stably transfected with the plasmid Epo-Luc plasmid. The EPO-hypoxia response element (HRE)-luciferase reporter plasmid contains three copies of the HRE consensus sequence from the promoter of the erythropoietin gene in the pGL3 vector: recombinant mouse IL-17 (R&D Systems, Minneapolis, MN, USA), rmIL-4 (Immunotools GmbH, Friesoythe, Germany) and rhVEGF-A (Immunotools GmbH), and GW9662 (Cayman Chem, Ann Arbor, MI, USA) and SR144528 (Cayman Chem). All other reagents were from Sigma Co (St Louis, MO, USA).

### M1 and M2 macrophage polarization assays

To study M1 differentiation induced by IL-17, serum-starved RAW264.7 macrophages were pre-incubated with VCE-004.8 for 18 h and exposed for an additional 24 h to recombinant mouse IL-17 (50 ng/ml) [29]. To study the M2 polarization, serum-starved macrophages were treated with VCE-004.8 or rmIL4 (40 ng/ml) [30] for 24 h. The cells were collected in PBS, and total RNA was extracted using the High Pure RNA Isolation Kit (Roche Diagnostics, Indianapolis, IN, USA).

### Transient transfection and luciferase assays

For Arg-1 gene promoter analysis, RAW264.7 cells were seeded in 24-well plates, and after 24 h, they were transiently transfected with the plasmid pGL3-mArg1 (Addgene, Cambridge, MA, USA) using Roti-Fect (Carl Roth, Karlsruhe, Germany) following the manufacturer’s specifications. To correct for transfection efficacy, 100 ng Renilla luciferase (pRL-CMV) was co-transfected. After stimulation, the luciferase activities were quantified using Dual-Luciferase Assay (Promega Co., Madison, WI, USA). For EPO-Luc transactivation experiments, NIH-3T3-EPO-luc cells were stimulated as indicated and the luciferase activity measured in the cell lysates after 6 h stimulation.

### Western blots

Cells were washed with PBS and proteins extracted in 50 μl of lysis buffer (50 mM Tris–HCl (pH 7.5), 150 mM NaCl, 10% glycerol, and 1% NP-40) supplemented with 10 mM NaF, 1 mM Na_3_VO_4_, 10 μg/ml leupeptine, 1 μg/ml pepstatin and aprotinin, and 1 μl/ml PMSF saturated. Thirty micrograms of proteins was boiled at 95 °C in Laemmli buffer and electrophoresed in 10% SDS/PAGE gels. Separated proteins were transferred to PVDF membranes (20 V for 30 min) and blocked in TBS solution containing 0.1% Tween 20 and 5% non-fat dry milk for 1 h at room temperature. Immunodetection of specific proteins was carried out by incubation with primary antibody against HIF-1α (1:1000; BD Biosciences, #610959 San Jose, CA, USA), HIF-2α (1:1000; Novus Biologicals, Littleton, USA), PHD1 (1:1000; Abcam, Cambridge, UK), PHD2 (1:1000; Abcam), PHD3 (1:1000; Abcam), OH-HIF-1α (1:1000; Cell Signaling, Danvers, MA, USA), PPARγ (1:1000), β-actin (1:10.000; Sigma), and arginase 1 (N20) (1:500; Santa Cruz, Dallas, TX, USA) overnight at 4 °C. After washing membranes, horseradish peroxidase-conjugated secondary antibody was added and detected by chemiluminescence system (GE Healthcare Europe GmbH).

### HIF-1α hydroxylation assay

MO3.13 cells were transfected with HA-PHD1 (Addgene), HA-PHD2 (Addgene), or HA-PHD3 (Addgene) as indicated. After 24 h of transfection, cells were stimulated with VCE-004.8, CBD, or DMOG at the concentrations indicated for 24 h. After that, PHDs were immunoprecipitated as described [31]. Recombinant human GST-HIF-1α protein (Abcam) and immunoprecipitated PHDs were incubated in the reaction buffer 50 mM Tris–HCl (pH 7.5), 1 mM DTT, 50 μM FeSO_4_, 5 mM ascorbate, and 200 μM oxoglutarate for 1 h at 30 °C, respectively. The prolyl hydroxylation reaction was stopped by adding Laemmli sample buffer and analyzed by immunoblot assays.

### Quantitative reverse transcriptase PCR

Total RNA (1 μg) was retrotranscribed using the iScript cDNA Synthesis Kit (Bio-Rad, Hercules, CA, USA) and the cDNA analyzed by real-time PCR using the iQTM SYBR Green Supermix (Bio-Rad) and a CFX96 Real-time PCR Detection System (Bio-Rad). GAPDH or HPRT genes were used to standardize mRNA expression in each sample. Gene expression was quantified using the 2−ΔΔCt method, and the percentage of relative expression against controls (untreated cells or mice) was represented. The primers used in this study are described in Table 1.

**Table 1:** Primers used in real-time PCR analysis

Genes	Forward (5′---3′)	Reverse (5′---3′)
TNF-α	CTACTCCCAGGTTCTCTTCAA	GCAGAGAGGAGGTTGACTTTC
IL-6	GTATGAACAACGATGATGCACTTG	ATGGTACTCCAGAAGACCAGAGGA3
CCL2	GGGCCTGCTGTTCACAGTT	CCAGCCTACTCATTGGGAT
CCL4	AGAAACAGCAGGAAGTGGGA	AACACCATGAAGCTCTGCGT
Arg-1	CTCCAAGCCAAAGTCCTTAGAG	AGGAGCTGTCATTAGGGACATC
Mrc1	CATGAGGCTTCTCCTGCTTCTG	TTGCCGTCTGAACTGAGATGG
IL-10	GGTTGCCAAGCCTTATCGGA	ACCTGCTCCACTGCCTTGCT
VEGF-A	CGAAGTGGTGAAGTTCATGGATG	TTCTGTATCAGTCTTTCCTGGTG
EPO	CTCCGAACAATCACTGCT	GGTCATCTGTCCCCTGTCCT
GAPDH	TGGCAAAGTGGAGATTGTTGCC	AAGATGGTGATGGGCTTCCCG
HPRT	ATGGGAGGCCATCACATTGT	ATGTAATCCAGCAGGTCAGCA

### PCR arrays

One microgram of RNA was transcribed to cDNA using the RT^2^ First-Strand Synthesis Kit (Qiagen, Hilden, Germany) and analyzed using the RT^2^ SYBR green qPCR master mix (Qiagen) and the Human Hypoxia Signaling Pathway Plus PCR array (Qiagen) in the case of HBMEC cell line studies. The expression profile of key genes involved in multiple sclerosis was studied using the Mouse Multiple Sclerosis RT^2^ Profiler PCR Array (Qiagen). Each array consists of 84 genes involved in hypoxia- or multiple sclerosis-related signaling, as well as 12 sequences to control for loading and cDNA quality. The fold change in gene expression was calculated using the 2−ΔΔCt method and five housekeeping genes for normalization following the manufacturer’s instructions. Each array was performed in triplicate.

### Determination of vascular endothelial growth factor (VEGF)

HMEC-1 and MO3.13 cells were treated with VCE-004.8 for 24 h. The culture supernatants were then collected and analyzed for VEGF by ELISA. The levels of VEGF were quantified with a Quantikine ELISA Human VEGF kit (R&D Systems, according to the manufacturer’s instructions.

### Angiogenesis assay

The angiogenesis assay with HUVEC cells was performed using the PrimeKit cryo (Essen Biosciences, Ann Arbor, MI, USA). Briefly, HUVEC CytoLight Green cells were co-cultured with Human Dermal Fibroblasts (NHDF) in 96-well plates and treated with VCE-004.8 or VEGFA for 7 days, and the tube formation process was monitored using integrated IncuCyte algorithms. The Matrigel assay was used to assess the formation of capillary-like structures in HBMEC. The cells were seeded over a uniform layer of Matrigel (Thermo Fisher Scientific, Waltham, MA, USA) in a 96-well plate in the presence of VEGFA (10 ng/ml) or VCE-004.8 (1 μM). After 5 h of treatment, tube formation was analyzed using a × 4 objective and a BD Pathway 855 Bioimager. ImageJ v1.45 software (http://rsbweb.nih.gov/ij/) was used to quantify the number of branches points of tubes.

### In vitro cell migration assays

The modulation of cell migration was analyzed by wound-healing assays. Briefly, MO3.13 cells were seeded in a 96-well Essen ImageLock plate (Essen BioScience) and were grown to confluence. After 24 h, the scratches were made using the 96-pin WoundMaker, followed by incubation of the cultures with rhVEGF (10 ng/ml) or conditioned media from HBMECs cells treated during 24 h with VCE-004.8 in the presence of 10 ng/ml of mitomycin C to block cell proliferation. Wound images were taken every 60 min for 18 h and the data analyzed by the integrated metric Relative Wound Density part of the live content cell imaging system IncuCyte HD (Essen BioScience).

### PGE_2_ release measurement in primary microglia cells

Rat microglial cells were purified from forebrains and cultured as previously described [32]. Cells were seeded on poly-d-lysine-coated plaques at a density of 50,000 cells/cm^2^ and maintained for 3 days in DMEM containing 5% horse serum. Then, the cells were incubated for 18 h with 0.5 μg/ml LPS, in the presence of different concentrations of VCE-004.8, and supernatants were collected. Supernatants were spun down at 2000 rpm for 10 min, 4 °C. PGE2 in microglia was analyzed and quantified by using the prostaglandin E2 EIA Kit (Cayman chemicals) following provider recommendations.

### Animals

All experiments were performed in strict accordance with EU and governmental regulations. The Ethics Committee on Animal Experimentation of the Instituto Cajal, Consejo Superior de Investigaciones Científicas (CSIC), approved all procedures described in this study. Handling of animals was performed in compliance with the guidelines of animal care set by the European Union guidelines 86/609/EEC, and the Ethics Committee on Animal Experimentation at the Cajal Institute (CSIC, Madrid) approved all the procedures described in this study (protocol number 96 2013/03 CEEA-IC). Measures to improve welfare assistance and clinical status as well as endpoint criteria were established to minimize suffering and ensure animal welfare. Briefly, wet food pellets are placed on the bed cage when the animals begin to develop clinical signs to facilitate access to food and hydration. Female C57BL/6 and SJL/J mice were purchased from Harlan (Barcelona, Spain) and housed in the animal facilities of the Cajal Institute under the following controlled conditions: 12-h light/dark cycle, temperature 20 °C (± 2 °C), and 40–50% relative humidity with free access to standard food and water.

### Determination of erythropoietin (EPO) induction in vivo in normal mice

Eight-week-old C57BL/6 male mice (Envigo, Barcelona, Spain) were dosed intraperitoneally (i.p.) with VCE-004.8 (10 mg/kg) for 3 weeks. Blood samples were taken under general anesthesia, and heparin plasma was collected. Samples were centrifuged for 20 min at 2000×*g* within 30 min of collection, and circulating levels of plasma EPO were quantified with a mouse EPO ELISA kit (R&D Systems) according to the manufacturer’s instructions. EPO values represent the mean ± SEM.

### Induction and assessment of EAE

EAE was induced in C57BL/6 female mice at 6–8 weeks of age by subcutaneous immunization with MOG_35–55_ (300 μg; peptide synthesis section, CBM, CSIC, Madrid, Spain) and 200 μg of *Mycobacterium tuberculosis* (H37Ra Difco, Franklin Lakes, NJ, USA) in a 1∶1 mix with incomplete Freund’s adjuvant (CFA, Sigma, #F5506). On the same day and 2 days later, mice were injected intraperitoneally (i.p.) with 200 ng of pertussis toxin (Sigma) in 0.1 ml PBS. Control animals (CFA) were inoculated with the same emulsion without MOG, and they did not receive pertussis toxin. Treatment started at day 8 post-immunization when animals showed the first symptoms of the disease and consisted in daily i.p. of VCE-004.8 (10 mg/kg) or vehicle alone (4% DMSO + 6.4% Tween 80 + phosphate-buffered saline) for the following 21 days (curative protocol). The mice were examined daily for clinical signs of EAE, and disease scores were measured as follows: 0, no disease; 1, limb tail; 2, limb tail and hind limb weakness; 3, hind limb paralysis; 4, hind limb and front limb paralysis; and 5, moribund and death. All animals were sacrificed at 28 days for further analysis.

### Theiler’s virus inoculation and clinical evaluation

TMEV-induced demyelinating disease (TMEV-IDD) was performed in SJL/J mice. Theiler’s virus (strain DA), given by Dr. Moses Rodriguez (Mayo Clinic, Rochester, NY, USA), was inoculated intracranially in the right cerebral hemisphere, with 2 × 10^6^ plaque forming units (pfu) in 30 μl of DMEM medium enriched with 5% fetal calf serum (FCS). Sham mice were inoculated with vehicle only (DMEM + 5% FCS). Sixty days after TMEV infection, mice were treated daily for 14 consecutive days with VCE-004.8 (10 mg/kg i.p.) or appropriate vehicle (4% DMSO + 6.4% Tween 80 + phosphate-buffered saline) (curative protocol). General health conditions and motor function of animals were periodically evaluated, from day 60, when animals showed their locomotor activity impaired, until day 75 post-infection. The screening for locomotor activity (LMA) was performed using an activity monitor system coupled to a Digiscan Analyser (Omnitech Electronics, Columbus, OH, USA). The data for the following variables of LMA for a session of 10 min were collected: horizontal activity, as the total number of beam interruptions of horizontal area sensors, and vertical activity, as the total number of beam interruptions in the vertical sensor.

### Tissue processing

Mice were anesthetized by i.p. administration of pentobarbital (50 mg/kg), and they were transcardially perfused with saline 0.9%. The spinal cord was obtained by extrusion with saline. Cervical spinal cord was immediately frozen and kept at − 80 °C for RT-PCR analysis; the remaining spinal cord was fixed in 4% paraformaldehyde in 0.1 M PBS, washed in 0.1 M PBS, cryoprotected with a 15% and then a 30% solution of sucrose in 0.1 M PBS, and frozen at − 80 °C. Free-floating thoracic spinal cord sections (15/30 μm thick: Leica Microsystems CM1900 cryostat, Barcelona, Spain) were then processed for immunohistochemistry.

### Immunohistochemistry

For immunofluorescence analysis, free-floating thoracic spinal cord sections were washed with 0.1 M PBS. Endogenous peroxidase activity was inhibited with 50% methanol and 1.66% hydrogen peroxide. The sections were blocked with 0.1% Triton X-100 and 5% animal serum and then incubated overnight at 4 °C in blocking buffer with the primary antibody. For IHC in 30-μm sections, microglia cells were stained with a rabbit anti-mouse Iba-1 antibody (1∶1000; Wako Chemical Pure Industry, Osaka, Japan) and a primary rat anti-mouse CD4 antibody (1∶1000; BD Pharmingen; San Diego, CA, USA) was used to detect CD4+ T cells (sections of 30 μm thick). In 15-μm sections, axons were stained with a neurofilament H antibody (1∶1000; Millipore; Temecula, CA, USA). After incubation with the primary antibody, the sections were rinsed with PBS three times for 10 min and then incubated for 1 h with the secondary antibody: biotinylated goat anti-rabbit (Iba-1), fluorescent goat anti-rabbit (neurofilament H), and biotinylated rabbit anti-rat (CD4). Myelin integrity was analyzed using the Hito CryoMyelinStain™ Kit (Gold phosphate complex Myelin Staining Kit) following manufacturer’s recommendation (Hitobiotech Corp., Kingsport, TN, USA).

### Inflammatory infiltrate analysis

Spinal cord slices were stained with hematoxylin-eosin (H&E) to analyze the infiltrates in the parenchyma. Inflammatory infiltrates were evaluated on a scale of 0 to 4, the score reflecting the number of infiltrates in the thoracic spinal cord sections. A score of 4 reflects the largest number of infiltrates with all the intermediate scores (1, 2, and 3) to define the increase in the density of infiltrates in the spinal cord tissue.

### Microscopy and image analysis

Six thoracic spinal cord sections per animal from at least six animals per group were taken. Staining was quantified using the ImageJ software (NIH; Bethesda, MD, USA). Sections were analyzed by immunofluorescence on a Leica TCS SP5 confocal microscope and with a Zeiss Axiocam high-resolution digital color camera for IHC.

### Data analysis

All the in vitro data are expressed as the mean ± SD. One-way ANOVA followed by the Tukey’s post hoc tests or unpaired two-tailed Student’s *t* test were used to determine the statistical significance. All the in vivo data are expressed as the mean ± SEM. Unpaired two-tailed Student’s *t* test for parametric analysis of two samples or Kruskal–Wallis test was used to determine the statistical significance in the case of non-parametric analysis. The level of significance was set at *p* <  0.05. Statistical analyses were performed using GraphPad Prism version 6.00 (GraphPad, San Diego, CA, USA).

## Results

### VCE-004.8 activated the HIF pathway

**Fig. 1:**
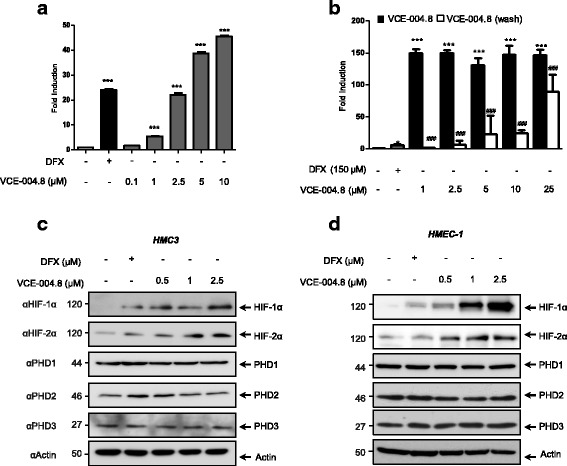
VCE-004.8 mediates HIF-1α stabilization. **a** NIH3T3-EPO-Luc cells were stimulated with VCE-004.8 at the doses indicated for 6 h and assayed for luciferase activity. **b** Cells were pre-treated with VCE-004.8 for 1 h and then washed or not with PBS and incubated in complete medium for 6 h. Fold induction relative to untreated cells is shown. Data represent the mean ± SD (*n* = 5). ***p < 0.001 VCE-004.8 or DFX-treated cells vs untreated cells (one-way ANOVA followed Tukey’s test). **c**, **d** HMC3 and HMEC-1 cells respectively were treated with VCE-04.8 and further analyzed for HIF-1α, HIF-2α, PHD1, PHD2, and PHD3 expression by immunoblot (*n* = 3)

**Fig. 2:**
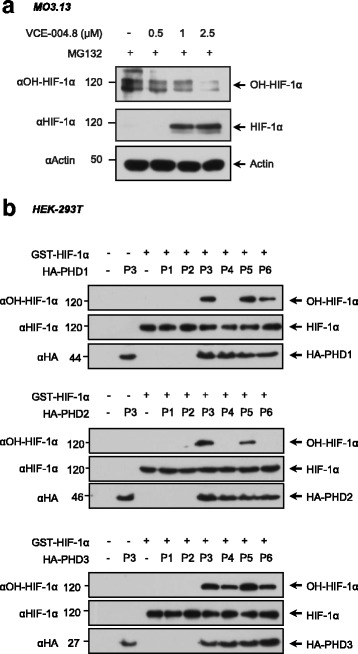
Effect of VCE-004.8 on PDH activity and HIF-1α hydroxylation and stabilization. **a** MO3.13 cells were treated with VCE-004.8 for 6 h in the presence of MG132 and the levels of hydroxylated HIF-1α and HIF-1α were determined by immunoblot (*n* = 3). **b** HEK-293T cells were transfected with HA-PHD1, HA-PHD2, or HA-PHD3 as indicated. After 24 h of transfection, cells were treated as follows: P1, non-transfected cells; P2, cells were transfected with PHDs and immunoprecipitated with IgG-HA; P3, cells were transfected with PHDs and immunoprecipitated with αHA; P4, cells transfected with PHDs, stimulated with VCE-004.8 (2.5 μM) and immunoprecipitated with αHA; P5, cells transfected with PHDs, stimulated with CBD (2.5 μM) and immunoprecipitated with αHA; P6, cells transfected with PHDs, stimulated with DMOG (1 mM) and immunoprecipitated with αHA. HIF prolyl hydroxylase activity was measured using GST-HIF-1α protein, and the levels of hydroxylated HIF-1α, HIF-1α, and PHDs were analyzed by immunoblot (*n* = 3)

VCE-004.8 is a dual PPARγ/CB_2_ agonist capable to prevent macrophage activation in vivo [28]. On the other hand, the structure of VCE-004.8 features the α-hydroxyenone and the α-aminoenone moieties typical of strong iron chelators [33] suggesting that it could modulate iron-sensitive molecular pathways like HIF. To evaluate this possibility, the transcriptional activity of the EPO gene, a process mainly regulated by HIF-1α/HIF-2α, was investigated. Figure 2a shows that VCE-004.8 strongly activated the EPO gene promoter in a concentration-dependent manner (*p* < 0.001 VCE-004.8 or DFX vs untreated). Next, to investigate the type of interaction with the HIF pathway, the induction of EPO-Luc activity was studied with washout experiments where VCE-004.8 was removed from the cell culture by washing the cells with PBS after 1 h of treatment, and EPO-Luc activity was then measured after an additional 5 h. Since this activity was greatly reduced 5 h after removal of VCE-004.8 from the cell medium, this compound was acting in a reversible manner (Fig. 1b). Next, we studied the effect of VCE-004.8 on the expression of both HIF-1α and HIF-2α at the protein level in different cell types. VCE-004.8 induced the stabilization of HIF-1α and HIF-2α in microglia (Fig. 1c) and endothelial microvascular (Fig. 1d) cell lines. In addition, VCE-004.8 could also induce HIF-1α/HIF-2α stabilization in the oligodendrocyte cell line MO3.13 (data not shown).

Conversely, VCE-004.8 did not affect the steady-state levels of any of the HIF prolyl hydroxylases (PHDs) analyzed, suggesting that the hypoxia mimetic activity of VCE-004.8 was the result of inhibition of the prolyl hydrolase functional activity. Accordingly, the inhibition of HIF-1α hydroxylation by VCE-00.8 paralleled the HIF-1α stabilization in MO13.3 cells preincubated with the proteasome inhibitor MG132 (Fig. 2a). To further identify which PHD is targeted by VCE-004.8, HEK293T cells were transfected with HA-tagged PHD1, PHD2, and PHD3 plasmids and next treated with VCE-004.8, CBD (negative control), and DMOG (positive control). After treatments, PHDs were immunoprecipitated and the prolyl hydrolase activity was measured by its capacity to hydroxylate GST-HIF-1α. VCE-004.8 was found to strongly inhibit PHD1 and PHD2 activity but did not affect PHD3 activity (Fig. 2b).

### Effect of VCE-004.8 on HIF-dependent gene expression, angiogenesis, and oligodendrocyte migration

**Fig. 3:**
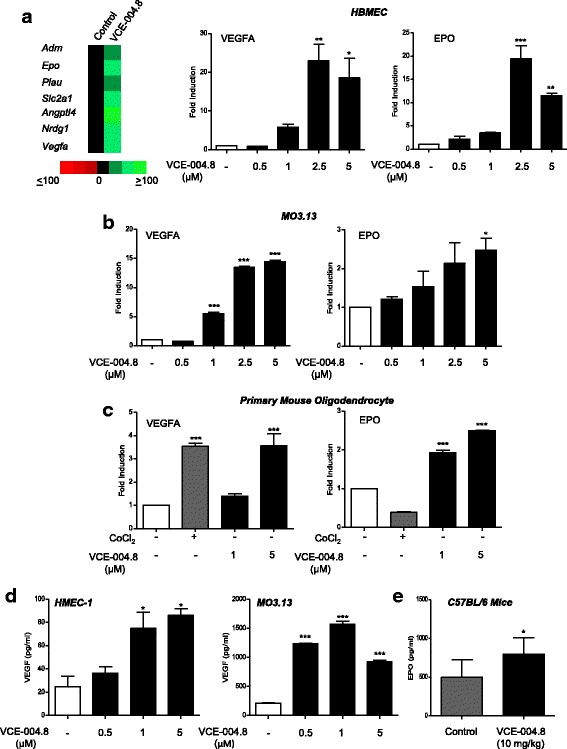
VCE-004.8 induces the expression of HIF-dependent genes. **a** Human brain microvascular endothelial cells were stimulated with VCE-004.8 (5 μM) for 12 h and the expression of genes involved in the human hypoxia signaling pathway determined by PCR array and qPCR in the case of VEGFA and EPO genes. Heat maps show the significantly upregulated (green) and downregulated (red) genes in VCE-004.8-treated cells compared with control. Data represent the mean ± SD (*n* = 3). *p < 0.05, **p < 0.01, ***p < 0.001 VCE-004.8-treated cells vs untreated cells (one-way ANOVA followed Tukey’s test). **b**, **c** The mRNA expression levels of VEGFA and EPO genes were quantified by qPCR in MO3.13 cells and primary mouse oligodendrocyte cells respectively. Data represent the mean ± SD (*n* = 3). *p < 0.05, ***p < 0.001 VCE-004.8-treated cells vs untreated cells (one-way ANOVA followed Tukey’s test). **d** VEGF production was determined by ELISA in the supernatants of HMEC-1 cells and M03.13 cells treated with VCE-004.8. Data represent the mean ± SD (*n* = 3). *p < 0.05, ***p < 0.001 VCE-004.8-treated cells vs untreated cells (one-way ANOVA followed Tukey’s test). **e** EPO levels were determined in plasma from C57BL/6 treated with VCE-004.8 (10 mg/kg) for 3 weeks. Plasma levels were increased after the treatment with VCE-004.8. Results are expressed as mean ± SEM (*n* = 6 animals per group). *p<0.05 vs untreated mice (unpaired two-tailed Student’s *t* test)

To investigate the effect of VCE-004.8 on the expression of HIF-dependent genes, HBMEC was treated with this compound and mRNA isolated and subjected to qRT-PCR for the expression of 83 genes involved in the hypoxia pathway (Fig. 3a). Fold up- or downregulation was calculated for each gene, and those whose expression was upregulated more than 10-fold were identified. VCE-004.8 clearly induced the expression of erythropoietin (*Epo*, *p* < 0.001 and *p* = 0.0018 VCE-004.8 vs untreated) and vascular endothelial growth factor (*Vegfa*, *p* = 0.0026 and *p* = 0.0117 VCE-004.8 vs untreated), which have a significant role in MS, and also the expression of other MS relevant genes such as adrenomedullin (*Adm*), plasminogen activator, urokinase (*Plau*), angiopoietin-like 4 (*Angptl4*), glucose transporter 1 or solute carrier family 2 member 1 (*Slc2a1*), and *N*-myc downstream regulated 1 (*Nrdg1*). Next, we evaluated the expression of VEGFA and EPO in oligodendrocytes. We found that VCE-004.8 also upregulated the expression of both HIF-dependent genes in MO3.13 cells [Fig. 3b (VEGFA, *p* < 0.001 VCE-004.8 vs untreated) (EPO, *p* = 0.0290 VCE-004.8 vs untreated)] and in murine primary oligodendrocytes [Fig. 3c (VEGFA, *p* = 0.0008 CoCl_2_ vs untreated; *p* = 0.0007 VCE-004.8 vs untreated) (EPO, *p* < 0.001 VCE-004.8 vs untreated)]. Moreover, VCE-004.8 also induced the release of VEGFA in both microvascular endothelial and oligodendroglial cells in a concentration-dependent manner [Fig. 3d (HMEC-1, *p* = 0.0401 and *p* = 0.0125 VCE-004.8 vs untreated) (MO3.13, *p* < 0.001 VCE-0048 vs untreated)] and increased the plasmatic levels of EPO in mice treated with the compound (Fig. 3e *p* = 0.0248 VCE-004.8 vs untreated).

**Fig. 4:**
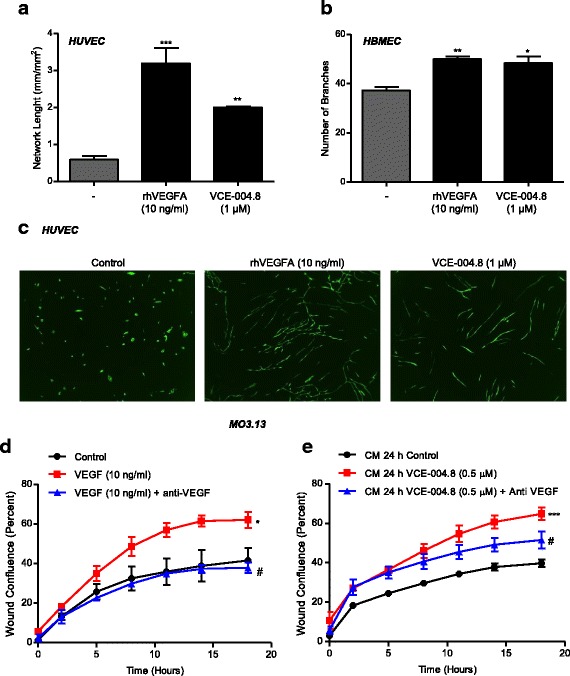
Functional consequences of VCE-004.8 on HIF-1α stabilization. **a** PrimeKit co-cultures were seeded on day 0 and the indicated concentration of rhVEGFA or VCE-004.8 was added on day 2. Tube formation was analyzed, and the results were plotted using the Incucyte FLR software in terms of network length on day 7 ± SD (*n* = 3). **p < 0.01, **p < 0.001 rhVEGFA- or VCE-004.8-treated cells vs untreated cells (one-way ANOVA followed Tukey’s test). **b** HBMECs were plated on Matrigel-coated cultured dishes and treated with VCE-004.8. Quantitative analysis of number of branch formation was performed using the ImageJ v1.45 software. Data represent the mean ± SD (*n* = 3). *p < 0.05, **p < 0.01 rhVEGFA- or VCE-004.8-treated cells vs untreated cells (one-way ANOVA followed Tukey’s test). **c** Representative images from the experiment described in **a** are shown (magnification × 4). **d**–**e** Scratch assay on MO3.13 cells treated with rhVEGFA in the presence of anti-VEGFA (**d**) or conditioned medium from HBMECs treated with VCE-004.8 in the absence or the presence of anti-VEGFA for 24 h (**e**). Results were plotted using the Incucyte FLR software in terms of percentage of wound confluence ± SD (*n* = 3) as a function on time. *p < 0.05, ***p < 0.001 vs control; ^#^*p* < 0.05 vs rhVEGFA- or VCE-004.8-treated cells (one-way ANOVA followed Tukey’s test)

Since VCE-004.8 induced the expression and release of VEGFA, we were interested in studying the potential activity of this compound in angiogenesis. We thought that VCE-004.8-triggered release of VEGFA could, in turn, induce angiogenesis in an autocrine manner. We investigated this possibility in two primary vascular endothelial cells, namely HUVEC and HMBEC, and using different methodology. Thus, in HUVEC co-cultured in a monolayer of primary fibroblasts, both rhVEGFA (10 ng/ml) and VCE-004.8 (1 μM) clearly induced an increase in the network of endothelial tubes [Fig. 4a (HUVEC, *p* < 0.001 rhVEGFA vs untreated; *p* = 0.001 VCE-004.8 vs untreated) and Fig. 4c (representative images)]. Next, HBMEC cells were cultured on Matrigel and similarly treated with rhVEGFA (10 ng/ml) or VCE-004.8 (1 μM). Figure 4b shows that both treatments increased the number of branch points as defined in the “Methods” section (HBMEC, *p* = 0.0063 rhVEGFA vs untreated; *p* = 0.0123 VCE-004.8 vs untreated).

We were also interested in exploring the effect of VCE-004.8 on real-time oligodendrocyte migration and the possible role of VEGFA in this activity. To this purpose, we first investigated the migratory capacity of differentiated MO13.3 cells in response to VEGFA. Cells were grown near to confluence in 96-well plates, a wound in the monolayer cells was then performed using a 96-pin WoundMaker, and cells were next treated with mitomycin C and stimulated with VEGFA in the absence or the presence of a neutralizing anti-VEGF mAb. We found that VEGFA induced migration in MO13.3 cells with the maximum healing activity at 18 h of culture (Fig. 4d *p* = 0.0252 VEGF vs untreated; *p* = 0.0199 VEGF + anti-VEGF vs VEGF). Next, we stimulated HBMEC with VCE-004.8 for 24 h, and the supernatants were collected (conditioned medium) and used to study the potential wound healing activity on MO13.3 cells. Figure 4e shows that CM from HBMEC clearly induced migration on MO3-13-scratched cells, and this effect was prevented only partially by the anti-VEGFA neutralizing antibody (*p* = 0.0009 VCE-004.8 vs untreated; *p* = 0.0146 VCE-004.8 + anti VEGF vs VCE-004.8). This result suggests that in addition to VEGFA, HBMEC also produces other factors that mediate oligodendrocyte migration.

### VCE-004.8 prevented IL-17-induced M1 polarization and LPS-induced COX-2 expression and enhanced arginase 1 expression in macrophages

**Fig. 5:**
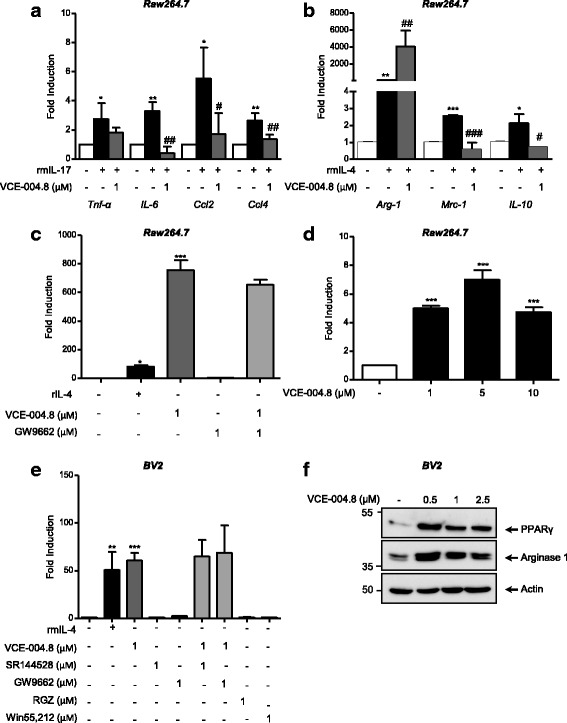
VCE-004.8 blunts IL-17-induced M1 polarization and induces Arg1+ expression. **a** RAW264.7 cells were pre-incubated with VCE.004.8 for 18 h and stimulated with IL-17 for 24 h. The mRNA expression for *Tnf-α*, *IL-6*, *Ccl2*, and *Ccl4* was quantified by qPCR and normalized versus GAPDH. Data represent the mean ± SD (*n* = 3). *p < 0.05, **p < 0.01 rmIL-17-treated cells vs untreated cells; ^#^*p* < 0.05, ^##^*p* < 0.01 rmIL-17 + VCE-004.8 vs rmIL-17-treated cells (one-way ANOVA followed Tukey’s test). **b** RAW264.7 cells were treated with VCE-004.8 in the presence or absence of rmIL-4 for 24 h. The mRNA expression for Arg-1, Mrc-1, and IL-10 was quantified by qPCR and normalized versus GAPDH. Data represent the mean ± SD (*n* = 3). *p < 0.05, **p < 0.01, ***p < 0.001 rmIL-4-treated cells vs untreated cells; ^#^*p* < 0.05, ^##^*p* < 0.01, ^###^*p* < 0.001 rmIL-4 + VCE-004.8 vs rmIL-4-treated cells (one-way ANOVA followed Tukey’s test). **c** RAW264.7 cells were treated with VCE-004.8, rmIL-4, or GW9662 for 24 h, and the expression levels of Arg-1 were determined by qPCR. Data represent the mean ± SD (*n* = 3). *p < 0.05 rmIL-4-treated cells vs untreated cells; ***p < 0.001 VCE-004.8 vs untreated cells (one-way ANOVA followed Tukey’s test). **d** RAW264.7 cells were transiently transfected with pGL3-mArg1 promoter/enhancer − 31/− 3810 and then stimulated with VCE-004.8 for 20 h and assayed for luciferase activity. Fold induction relative to transfected cells untreated is shown. Data represent the mean ± SD (*n* = 3). ***p < 0.001 VCE-004.8-treated cells vs untreated cells (one-way ANOVA followed Tukey’s test). **e** Serum-starved BV2 cells were treated with VCE-004.8, rmIL-4, SR144528, GW9662, RGZ, or WIN 55,212-2 for 24 h, and the expression levels of Arg-1 were determined by qPCR. Data represent the mean ± SD (*n* = 3). *p < 0.05 rmIL-4-treated cells vs untreated cells; ***p < 0.001 VCE-004.8 vs untreated cells (one-way ANOVA followed Tukey’s test). **f** BV2 cells were treated with VCE-004.8 at the concentrations indicated and further analyzed for PPARγ and arginase 1 expression by immunoblot

It has been shown that Arg-1 gene expression can be upregulated by the HIF pathway and that HIF-2α mRNA is expressed in M2-polarized macrophages and activates Arg-1 expression [34]. Since Arg-1 is considered a marker for M2a polarization, we were interested in studying the effect of VCE-004.8 on macrophage polarization. Treatment of RAW264.7 macrophages with IL-17 promoted their polarization towards a pro-inflammatory M1 phenotype, as shown by increased expression of M1 markers like TNFα, IL-6, Ccl2, and Ccl4. Exposing RAW264.7 macrophages to VCE-004.8 strongly inhibited the induction of M1 markers by IL-17 [Fig. 5a (TNFα, *p* = 0.0313 IL-17 vs untreated) (IL-6, *p* = 0.0034 IL-17 vs untreated; *p* = 0.002 IL-17 + VCE004.8 vs IL-17) (Ccl2, *p* = 0.0174 IL-17 vs untreated; *p* = 0.0337 IL-17 + VCE-004.8 vs IL-17) (Ccl4, *p* = 0.0332 IL-17 vs untreated; *p* = 0.0109 IL-17 + VCE-004.8 vs IL-17)]. Interestingly, VCE-004.8 enhanced the expression of Arg-1 in IL-4-treated cells without affecting the expression of other M2 markers such as Mcr-1 and IL-10 [Fig. 5b (Arg-1, *p* = 0.025 IL-4 vs untreated; *p* = 0.0085 IL4 + VCE-004.8 vs IL-4) (Mrc-1, *p* = 0.0005 IL-4 vs untreated; *p* < 0.0001 IL-4 + VCE-004.8 vs IL-4) (IL-10, *p* = 0.0217 IL-4 vs untreated; *p* = 0.0146 IL-4 + VCE-004.8 vs IL-4)]. VCE-004.8 also induced a strong expression of Arg-1 in the absence of IL-4, and this activity was not prevented by the PPARγ antagonist GW9662 (Fig. 5c *p* = 0.032 IL-4 vs untreated; *p* < 0.001 VCE-004.8 vs untreated). To further investigate if the effect of VCE-004.8 on Arg-1 expression was exerted at the transcriptional level, RAW264.7 cells were transfected with a plasmid containing the *Arg1* promoter fused to luciferase gene. Figure 5d shows that VCE-004.8 induced the transactivation of the *Arg1* promoter (*p* < 0.001 VCE-004.8 vs untreated). VCE-004.8 also upregulated Arg-1 mRNA expression in microglia cells through PPARγ- and CB_2_-independent pathways (Fig. 5e *p* = 0.0036 IL-4 vs untreated; *p* = 0.0006 VCE-004.8 vs untreated) and increased the steady-state protein expression of PPARγ and Arg-1 (Fig. 5f). In addition, neither rosiglitazone, a PPARγ agonist, nor WIN 55,212-2, a dual CB_1_/CB_2_ agonist, induces *Arg1* mRNA expression (data not shown).

To further whether VCE-004.8 exerts anti-inflammatory effects in primary cells, microglia cells were pre-incubated with VCE-004.8 for 30 min and then stimulated with or without LPS (10 ng/ml) for 24 h. As a result, an increase in the production of PGE_2_ was observed in LPS-treated cells compared to unstimulated cells. Treatment with VCE-004.8 prior to stimulation with LPS resulted in significant decrease of PGE_2_ release when compared with LPS (considered as 100%). Significant reductions in the levels of PGE_2_ were evident starting from the concentration of 1 μM (*p* < 0.05), and a pronounced decrease was observed at the conc. of 10 μM (mean *p* < 0.001) (Additional file 1: Figure S1A). PGE_2_ synthesis occurs during neuroinflammation through the enzymatic action of COX-2. Consequently, we determined whether the effect of VCE-004.8 on PGE_2_ is mediated by direct inhibition of COX-2 upregulation in LPS-treated cells. We found that VCE-004.8 strongly inhibited the expression of COX-2, another M1 marker, induced by LPS in primary microglia cells (Additional file 1: Figure S1B).

### VCE-004.8 attenuates the clinical severity and neuropathology in EAE and TMEV

**Fig. 6:**
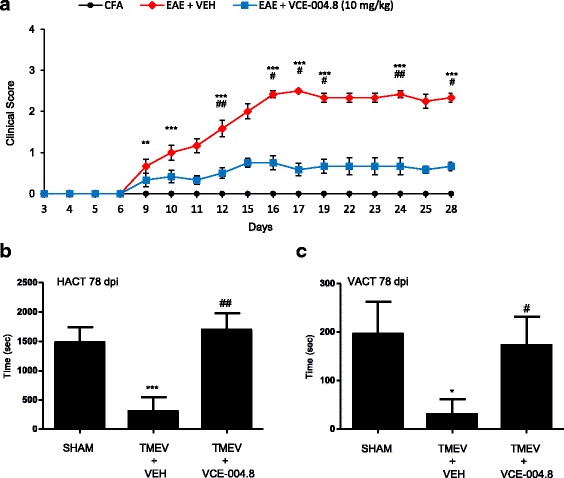
VCE-004.8 attenuates the clinical severity and neuropathology in EAE and TMEV models. **a** VCE-004.8 significantly ameliorated the clinical signs and progression of EAE. Results are expressed as mean ± SEM (*n* = 6 animals per group). **p < 0.01 ***p < 0.001 EAE + VEH vs CFA; ^#^*p* < 0.05, ^##^*p* < 0.01, ^###^*p* < 0.001 EAE + VCE-004.8 vs EAE + VEH (one-way ANOVA followed Tukey’s test). The actimeter test showed a significant reduction of the horizontal exploratory activity (**b**) and of the vertical activity (**c**) of TMEV group at 78 dpi. The treatment with VCE-004.8 ameliorated the motor deficits in mice infected with Theiler’s virus. *p < 0.05 **p < 0.01 TMEV + VEH vs Sham; ^#^*p* < 0.05, ^##^*p* < 0.01 TMEV + VCE-004.8 vs TMEV + VEH (non-parametric Kruskal–Wallis test)

Since VCE-004.8 is a multifunctional compound acting on a series of critical targets in the MS physiopathology, we tested its efficacy on different murine models of neuroinflammation and demyelination. The therapeutic potential of VCE-004.8 was first evaluated in EAE, performing the treatments at an early stage of the disease since mice received the first injection of VCE-004.8 (10 mg/kg) at day 6 p.i. (post-immunization). Subcutaneous immunization with MOG_35–55_ induced EAE in all mice that received the vehicle alone. All vehicle-treated mice developed a disease that peaked by day 17 p.i. and maintained at day 28 p.i. By contrast, the clinical manifestations of EAE were attenuated in mice receiving daily injections of VCE-004.8 and the disease peaked on day 23 p.i. not reaching a score of 1 throughout the course of the experiment (day 6–day 28) (Fig. 6a *p* < 0.001 EAE + VEH vs CFA; *p* < 0.001 EAE + VCE-004.8 vs EAE + VEH at day 28).

The effect of VCE-004.8 was next investigated in another model of MS, assumed to mimic primary progressive MS. Hence, 60 days after TMEV infection, the mice were treated with VCE-004.8 (10 mg/kg) or vehicle for 14 days. Then, mice were examined, and clinical scores were assigned to all of them. Motor activity was assessed in an activity cage, and horizontal activity and vertical activity were analyzed [Fig. 6b (HACT, *p* = 0.01 TMEV + VEH vs SHAM; *p* = 0.003 TMEV + VCE-004.8 vs TMEV + VEH); Fig. 6c (VACT, *p* = 0.034 TMEV + VEH vs SHAM; *p* = 0.050 TMEV + VCE-004.8 vs TMEV + SHAM)]. Sham animals exhibited normal activity levels: 1498 bean interruptions (average) for horizontal activity and 195 times (average) standing up for vertical activity. TMEV infection dramatically reduced both horizontal and vertical activities to very low levels. Strikingly, treatment with VCE-004.8 completely abrogated the decreased motor activity, recovering motor activities to normal levels.

**Fig. 7:**
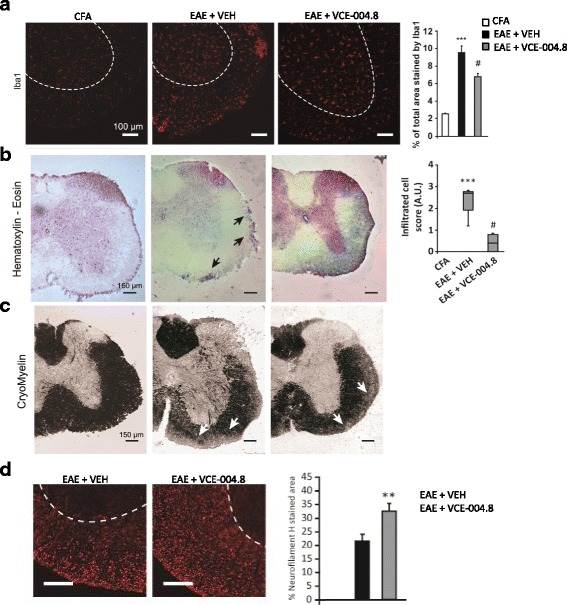
VCE-004.8 significantly reduces microglial reactivity and infiltration of inflammatory cells and preserves myelin structure in EAE animals. Cross-sectional images of thoracic–lumbar spinal cord cross-sections of 30 μm thick, in which immunofluorescence with anti-Iba1 (**a**), H-E (**b**), cryomyelin (**c**), and neurofilament H staining (**d**) immunohistochemistry was performed. Treatment with VCE-004.8 reduces the number of cell infiltrates (**b**, arrows) and a significant decrease in microglial reactivity (**a**). The quantifications are shown as means ± SEM, and significance was determined by Kruskal–Wallis non-parametric test. ***p < 0.001 vs CFA; ^#^*p* < 0.05 ^###^*p* < 0.001 vs EAE + VEH. Scale bar: 150 μm (**a**), 100 μm (**c** and **e**). *p < 0.05 unpaired two-tailed Student’s *t* test (**d**)

**Fig. 8:**
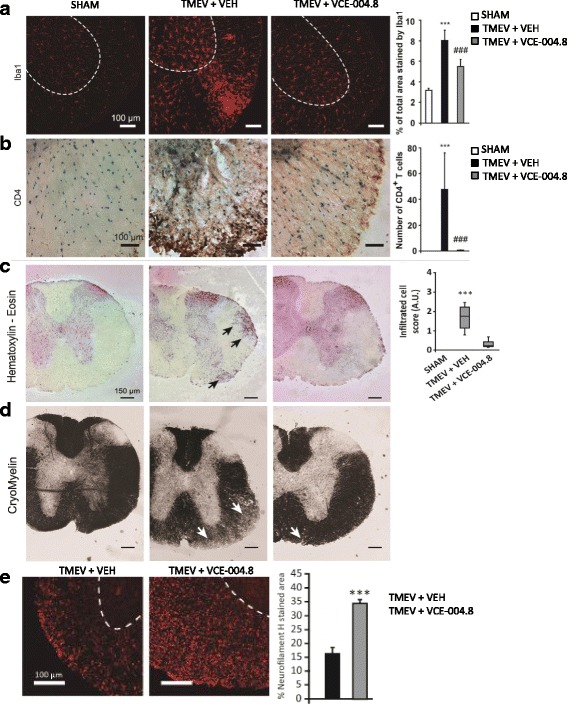
VCE-004.8 significantly reduces microglial reactivity and infiltration of inflammatory cells and preserves myelin structure in TMEV-infected mice. Cross-sectional images of thoracic–lumbar spinal cord cross-sections of 30 μm thick, in which immunofluorescence with anti-Iba1 (**a**), anti-CD4 (**b**), H-E (**c**), cryomyelin (**d**), and neurofilament H staining (**e**) immunohistochemistry was performed. Treatment with VCE-004.8 reduces the number of CD4+ T cells, cell infiltrates (**c**, arrows), and a significant decrease in microglial reactivity (**a**). The quantifications are shown as means ± SEM, and significance was determined by Kruskal–Wallis non-parametric test. ***p < 0.001 vs Sham; ^##^*p* < 0.01, ^###^*p* < 0.001 vs TMEV + VEH. Scale bar: 150 μm (**a**), 100 μm (**c** and **e**). ***p < 0.001 unpaired two-tailed Student’s *t* test (**d**)

**Fig. 9:**
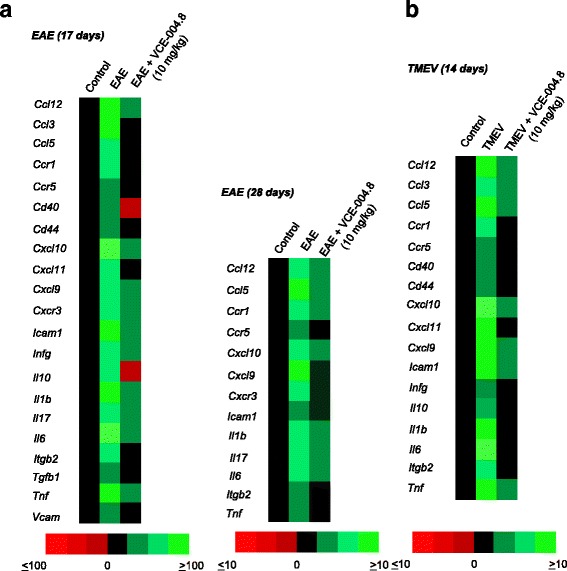
VCE-004.8 modulates gene expression in the spinal cord of EAE and TMEV-infected mice. Animals were treated as indicated, and total RNA was isolated. One microgram of RNA was retrotranscribed, and the resulting cDNA was analyzed in a mouse MS PCR array. Heat maps represent genes with significant upregulation (green) or downregulation (red) in EAE model (**a**) and TMEV-infected mice (**b**)

To study whether VCE-004.8 was able to target neuroinflammation in EAE and TMEV mice, microgliosis, leucocyte infiltration, and the expression of different inflammatory mediators were evaluated in the spinal cord of infected animals. Histopathological analysis showed that the extensive microglia/macrophage activation in the spinal cord of EAE and TMEV mice evidenced by Iba-1 staining was dramatically reduced by VCE-004.8 in both EAE (Fig. 7a *p* < 0.001 EAE + VEH vs CFA; *p* = 0.026 EAE + VCE-004.8 vs EAE + VEH) and TMEV (Fig. 9a *p* < 0.001 TMEV + VEH vs SHAM; *p* = 0.0017 TMEV + VCE-004.8 vs TMEV + VEH) mice. In addition, EAE (Fig. 7b *p* < 0.001 EAE + VEH vs CFA; *p* < 0.001 EAE + VCE-004.8 vs EAE + VEH) and TMEV (Fig. 8c *p* = 0.0058 TMEV + VEH vs SHAM) mice showed infiltration of immune cells into the spinal cord that was reduced by VCE-004.8 as shown by hematoxylin–eosin staining. Interestingly, VCE-004.8 treatment reduced the number of CD4+ T cells in the spinal cord of TMEV mice (Fig. 8b *p* < 0.001 TMEV + VEH vs SHAM; *p* < 0.001 TMEV + VCE-004.8 vs TMEV + VEH).

Demyelination and axonal damage are considered the histopathological hallmark of MS. Therefore, we studied the effect of VCE-004.8 on myelin expression and axonal damage in EAE and TMEV mice. To determine the extent of demyelination, myelin was stained using a gold phosphate complex myelin staining kit in stained preparations, and myelin is intensely black, so white matter is well differentiated from gray matter. An intense demyelination was found in the spinal cord of EAE and TMEV mice and significantly prevented by the treatment with VCE-004.8 (Figs. 7c and 8d). To further evaluate the potential of VCE-004.8 as a neuroprotective agent, we compared the axonal integrity in EAE and TMEV mice that received the vehicle alone or VCE-004.8 by assessing neurofilament H labelling. In representative coronal thoracic spinal cord sections, neurofilament H staining highlights the axonal disorganization in EAE and TMEV control mice that prevented by the treatment with VCE-004.8 (Fig. 7d *p* = 0.0119 EAE + VCE-004.8 vs EAE + VEH and Fig. 8e *p* < 0.001 TMEV + VCE-004.8 vs TMEV + VEH).

Finally, to understand the molecular mechanisms underlying the beneficial effects of VCE-004.8 in EAE and TMEV mice, the impact of VCE-004.8 on gene expression was comparatively evaluated in the spinal cord of control, sick, and treated animals. mRNA was isolated from the spinal cord, and the expression of 83 genes involved in MS physiopathology was analyzed by qRT-PCR. Fold upregulation was calculated for each gene in the spinal cord of EAE mice at the peak of the disease, and we found that a strong upregulation of proinflammatory mediators was observed, including chemokines such as *Ccl12*, *Ccl3*, *Ccl5*, *Cxcl10*, *Cxcl11*, and *Cxcl9*; chemokines receptors such as *Ccr1*, *Ccr5*, and *Cxcr3*; cytokines such as *Infg*, *Il1b*, *Il6*, *Tnf*, *Il17*, *Tgfb1*, *Tgfb2*, and *Il10*; adhesion molecules such as *Icam1* and *Vcam*; and other markers like *CD40*, implicated in immune cell migration, activation, and differentiation, and *CD44*. The expression of all those makers was significantly reduced or even abolished in mice treated with VCE-004.8. After 28 days of disease, many of the proinflammatory markers continued to be upregulated and their expression was also prevented by VCE-004.8 (Fig. 9a). We also performed a similar analysis in TMEV mice, where a strong upregulation of pro-inflammatory markers was also identified, with a similar, but not identical, pattern of neuroinflammation in both models. Again, VCE-004.8 inhibited the expression of inflammatory markers in TMEV mice (Fig. 9b).

## Discussion

Natural products, including phytocannabinoids, have been successfully used for the development of semisynthetic derivatives with improved bioactivities and clinical profile compared to the parent lead structure [35]. Thus, cannabidiol (CBD) is a poor PPARγ agonist, unable to bind CB_2_ and to activate the HIF pathway [36–38], but oxidation of its resorcinol core to a quinoid system increases PPARγ binding, while the introduction of an additional nitrogen substituent, a benzylamino group in VCE-004.8, improves stability and induces CB_2_ binding [28, 36]. We have now discovered that these modifications also induce hypoxia mimetic activity in VCE-004.8 and that the overall bioactivity profile of this compound is endowed with significant potential of development for the management of MS.

The mechanism by which O_2_ controls HIF-1α and HIF-2α stabilization has been revealed by the identification of PHDs, which are non-heme iron-containing dioxygenases requiring molecular oxygen and 2-oxoglutarate to hydroxylate HIF-1α and HIF-2α [10]. Under normoxic conditions, hydroxylated HIF is ubiquitinated by an E3-ubiquitin ligase and prepared for degradation by the 26S proteasome [39]. The development of PHDs inhibitors for the treatment of a wide number of diseases has recently attracted considerable attention [10, 40, 41]. We have discovered that the semi-synthetic cannabinoid VCE-004.8 inhibits HIF-1α hydroxylation and the activity of PHD1 and PHD2. VCE-004.8 is endowed with structural features typical of iron chelators, like the hydroxy- and the aminoenone motifs, but, surprisingly, we found that VCE-004.8 does not mimic the activity of the ion chelator DFX in vitro assays of PDH2 activity (data not shown). It has been described that PHDs may also undergo posttranslational modifications affecting their activity. For instance, PHD1 is phosphorylated in serine 130 by cyclin-dependent kinases 2, 4, and 6, and PHD2 is phosphorylated at serine 125 by P70S6K [42, 43], and VCE-004.8 could modulate these processes, a working hypothesis for currently ongoing activities. On the other hand, the pleiotropic profile of this compound towards some important endpoints of clinical research on MS prompted us to investigate if VCE-004.8 could indeed qualify for further development in this therapeutic area.

Microglial activation has been studied extensively in MS patients and in animal models. In addition, infiltrating proinflammatory macrophages (M1) have also been identified as major effectors of demyelination in MS, with the requirement for lymphocytes being in some cases negligible and macrophages being the sole mediators of demyelination [44]. Conversely, M2-polarized macrophage/microglia promote neuronal survival, neurite outgrowth, and OPC differentiation [45–48]. We found that VCE-004.8 blunts M1 polarization, an observation in agreement with previous reports showing that PPARγ and CB_2_ ligand activators inhibited M1 polarization [29, 49]. However, VCE-004.8 failed to induce a complete M2 polarization in the presence of IL-4, rather strongly upregulating the expression on Arg-1. This is a surprising finding, since PPARγ ligands affect macrophage M2 differentiation [49]. A possible explanation is that VCE-004.8, by acting as a partial PPARγ agonist, induces a different gene expression profile compared to full PPARγ agonists like glitazones [28, 50]. It is also possible that, by activating HIF-1α, VCE-004.8 may block full M2 differentiation, allowing only the expression of Arg-1 through HIF-2α, which is also stabilized in VCE-004.8-treated microglia cells [34, 51]. Arg-1 and inducible NO synthase (iNOS) are arginine-metabolizing enzymes that compete for the use of l-arginine and exert opposite functions in different pathological conditions including MS [52–54]. In addition, arginase activity is increased in plasma of MS patients responding to interferon-β1b therapy [55]. VCE-004-8-induced Arg-1 activity might therefore counteract the proinflammatory effects of iNOS. Interestingly, it has recently been shown the existence of a crosstalk pathway between l-arginine and the l-tryptophan pathway that plays an important role in the pathogenesis of MS. l-tryptophan is metabolized by indoleamine 2–3 dioxygenase 1 (IDO-1), whose activity in dendritic cells is strictly dependent on Arg-1 expression [56]. Thus, it is possible that Arg-1 upregulated by VCE-004.8 could induce tolerogenic dendritic cells (tDC) in the CNS through IDO-1 activation. tDC are being investigated in clinical trials for the treatment of several autoimmune diseases including MS [57]. Interestingly, it has been shown that hypoxia upregulates IDO-1 expression in the hippocampus [58]. Therefore, the potential role of VCE-004.8 on IDO-1 activation and the induction of tDC through l-tryptophan metabolism warrants further research.

Previous studies have shown that HIF-1α is also implicated in T cell differentiation and function. Indeed, HIF-1α enhances Th17 differentiation by activating the retinoid-related orphan receptor γt that interacts with p300 to induce the transcriptional activity of the IL-17 promoter [59]. However, another study has shown that pharmacological activation of the HIF pathway promotes the differentiation of Treg cells [60]. Indeed, CB_2_ and PPARγ ligand activators have both been reported to inhibit Th17 differentiation in the EAE model and in peripheral T cells from MS patients, respectively [61, 62]. Our results showed that IL-17 gene expression is induced in the spinal cord of EAE mice and is inhibited by the treatment with VCE-004.8. In addition, VCE-004.8 could inhibit CD3-induced IL-17 promoter transactivation in T cells (unpublished results), making it possible that CB_2_/PPARγ may abrogate the effect of HIF-1α on Th17 cell differentiation.

Therapeutic anti-inflammatory strategies for the treatment of MS involve neutralization of molecules involved in chemotaxis, adhesion, and migration of inflammatory cells to the CNS. The immunomodulatory activity of VCE-004.8 in EAE and TMEV mice was evidenced by the inhibition of several inflammatory chemokines (*Ccl12*, *Ccl3*, *Ccl5*, *Cxcl10*, *Cxcl11*, and *Cxcl9*), chemokines receptors (*Ccr1*, *Ccr5*, and *Cxcr3*), and cytokines (*Infg*, *Il1b*, *Il6*, *Tnf*, and *Il17*). In addition, VCE-004.8 inhibited the expression of adhesion molecules such as VCAM and ICAM-1. Chemokines and their receptors are essential for monocyte and lymphocyte migration into the CNS and therefore play a key role in the pathogenesis of MS. We have previously shown that WIN 55,212-2, a CB_1_/CB_2_ agonist, inhibited ICAM-1 and VCAM-1 expression in brain endothelial cells acting partially through the PPARγ pathway [63]. Therefore, it is likely that the potent anti-inflammatory activity of VCE-004.8 is mediated by the modulation of CB_2_ and PPARγ.

VEGF is a prototypical neurovascular signal that regulates vascular and neuronal functions. This factor has multiple direct beneficial effects on various neural cell types including OPCs and oligodendrocytes [64]. However, VEGF is a double-edged sword in MS and in other diseases like neuromyelitis optica and macular degeneration [64]. Thus, low levels of VGEF are necessary for endothelial cell survival and integrity of the blood-brain barrier (BBB), but high levels induce BBB dysfunction and are detrimental for CNS vascular homeostasis. Accordingly, blocking the aberrant expression of VEGF in EAE reduces neuroinflammation and demyelination and suppresses angiogenesis [65]. Dissociation of the neurogenic and neuroprotective activities of VEGF from its effects on vascular permeability might represent a therapeutic avenue for the development of novel therapies for the treatment of different MS subtypes [11]. It is therefore remarkable that VCE-004.8 induced VEGF secretion by oligodendrocytes and brain vascular endothelial cells without any apparent sign of BBB dysfunction in EAE and TMEV mice. The VEGF effects on BBB disruption could be prevented by EPO, which is also induced by VCE-004.8 [66, 67]. Moreover, VCE-004.8-mediated PPARγ activation may also contribute to maintain BBB integrity, since PPARγ agonists protect the BBB in models of brain stroke [68]. Indeed, it has been shown that CBD, the precursor of VCE-004.8, prevents BBB dysfunction through a PPARγ-dependent pathway [69]. Last, we cannot discard the view that VCE-004.8, as HIF PHDs inhibitor, may also induce the expression of endogenous VEGF-B and the spliced isoform VEGF_165_b that are non-angiogenic but retains the neuroprotective activity of VEGF [70].

Also remarkable is our finding that VCE-004.8 strongly induced the expression of other HIF-dependent genes beneficial for MS, like adrenomedullin (*Adm*), in HBMEC. *Adm* is important in endothelial cell survival and regulation of BBB permeability, is protective towards white matter, modulates oligodendrocytes function, and exerts an overall protective activity on EAE [71–73]. Angiopoietin-like 4 (*Angptl4*) and *N*-myc downregulated gene 1(*Nrdg1*) were upregulated by 145.6- and 25.2-fold, respectively, in VCE-004.8-treated cells. Angptl4 modulates BBB dysfunction in ischemic stroke and is neuroprotective [74]. On the other hand, Nrdg1 plays a role on oligodendrocytes survival and the gene is methylated and represses in the normal-appearing white matter of postmortem MS patients [75].

## Conclusions

In conclusion, we provide evidence that VCE-004.8 is a promising small molecule to modulate relevant MS targets, being endowed with PPARγ and CB_2_-mediated neuroinflammatory activity, and may enhance remyelination by inhibiting PHD activity and inducing neuroprotective factors such as VEGF and EPO.

## Additional file

Additional file 1: **Figure S1.** VCE-004.8 inhibits COX-2 expression and PGE2 release in LPS-stimulated rat primary microglia cells. (A) PGE2 released was analyzed in the supernatants from primary microglia cells treated as in A. Data represent the mean ± SD (*n* = 3). *p < 0.05 **p < 0.01, ***p < 0.001 (one-way ANOVA followed Tukey’s test). (B) Primary microglia cells were pre-treated with increasing concentrations of VCE-004.8, then stimulated with LPS for 24 h and the expression of COX-2 analyzed by immunoblot (*n* = 2). (PPTX 384 kb)
