# Supplementary material for: Hypoxia mimetic activity of VCE-004.8, a cannabidiol quinone derivative: implications for multiple sclerosis therapy
Source: J Neuroinflammation. 2018 Mar 1;15:64. doi: 10.1186/s12974-018-1103-y (PMC5831753; doi:10.1186/s12974-018-1103-y)
Supplement: Supplementary file 1 — Figure S1. VCE-004.8 inhibits COX-2 expression and PGE2 release in LPS-stimulated rat primary microglia cells. (A) PGE2 released was analyzed in the supernatants from primary microglia cells treated as in A. Data represent the mean ± SD (n = 3). *p < 0.05 **p < 0.01, ***p < 0.001 (one-way ANOVA followed Tukey’s test). (B) Primary microglia cells were pre-treated with increasing concentrations of VCE-004.8, then stimulated with LPS for 24 h and the expression of COX-2 analyzed by immunoblot (n = 2). (PPTX 384 kb) [file 12974_2018_1103_MOESM1_ESM.pptx]

## Slide 1
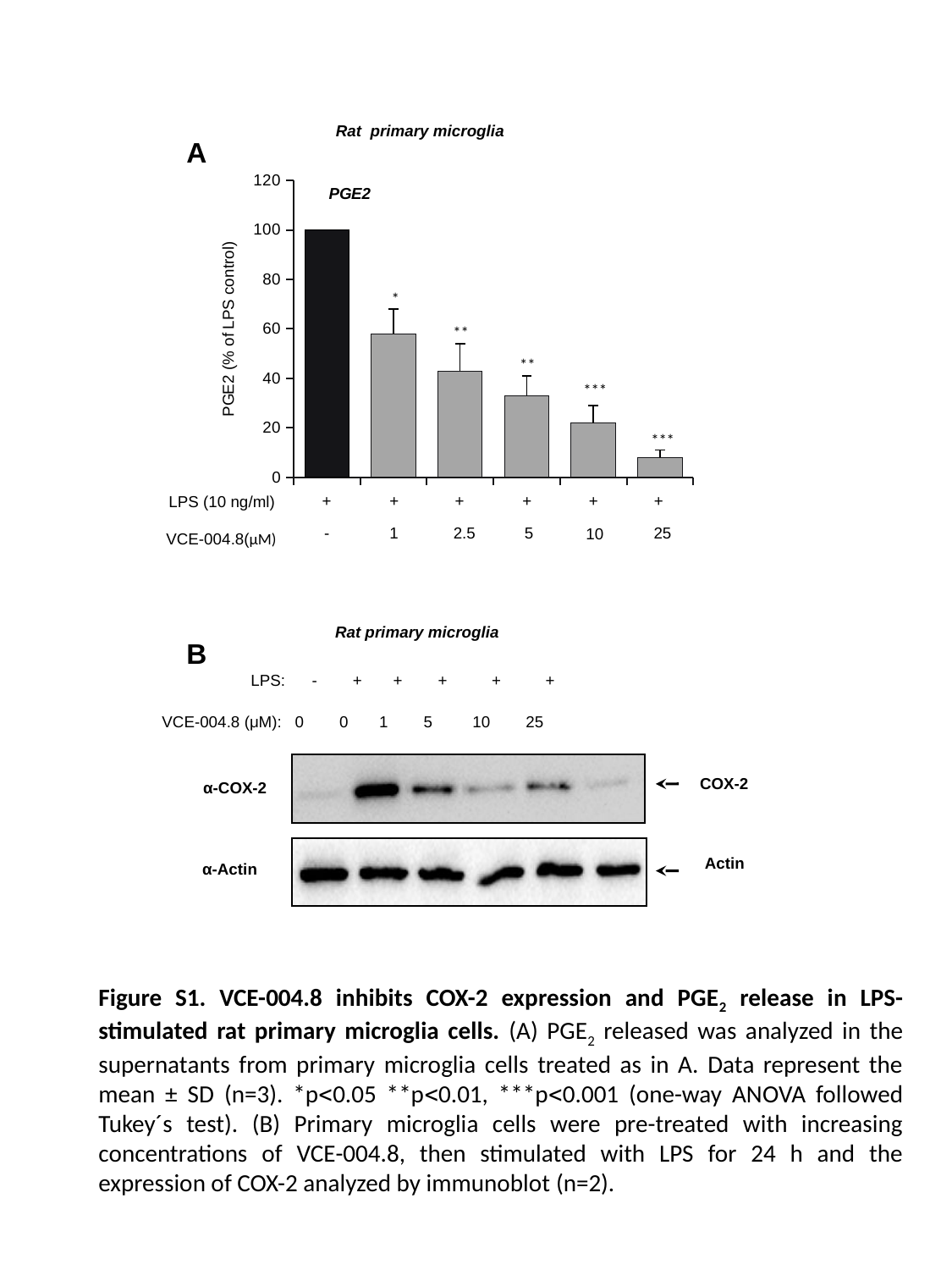

Rat primary microglia
A
### Chart: PGE2
| Category | |
|---|---|*
**
**
***
***
+
+
+
+
+
+
LPS (10 ng/ml)
-
1
2.5
5
25
10
VCE-004.8(µM)
Rat primary microglia
B
LPS: - + + + + +
 VCE-004.8 (μM): 0 0 1 5 10 25
COX-2
α-COX-2
Actin
α-Actin
Figure S1. VCE-004.8 inhibits COX-2 expression and PGE2 release in LPS-stimulated rat primary microglia cells. (A) PGE2 released was analyzed in the supernatants from primary microglia cells treated as in A. Data represent the mean ± SD (n=3). *p0.05 **p0.01, ***p0.001 (one-way ANOVA followed Tukey´s test). (B) Primary microglia cells were pre-treated with increasing concentrations of VCE-004.8, then stimulated with LPS for 24 h and the expression of COX-2 analyzed by immunoblot (n=2).
